# Utility and Necessity of Repeat Testing of Critical Values in the Clinical Chemistry Laboratory

**DOI:** 10.1371/journal.pone.0080663

**Published:** 2013-11-19

**Authors:** Aijun Niu, Xianxia Yan, Lin Wang, Yan Min, Chengjin Hu

**Affiliations:** 1 Department of Laboratory Medicine, the General Hospital of Jinan Military Region, Jinan, China; 2 Department of Laboratory Medicine, Mental Heath Center of Shandong Province, Jinan, China; University of California Los Angeles, United States of America

## Abstract

**Context:**

Routine repeat testing of critical values is a long-standing practice in many clinical laboratories; however, its usefulness and necessity remain to be empirically established and no regulatory requirements yet exist for verification of the critical value results obtained by repeat analysis.

**Objective:**

To determine whether repeat testing of critical values is useful and necessary in a clinical chemistry laboratory.

**Methods:**

A total of 601 chemistry critical values (potassium, n = 255; sodium, n = 132; calcium, n = 108; glucose, n = 106) obtained from 72,259 routine clinical chemistry specimens were repeat tested. The absolute value and the percentage of difference between the two testing runs were calculated for each of the four critical values and then compared with the allowable error limit put forth in the College of American Pathologists (CAP).

**Results:**

Among the repeat data for the 601 critical values, a total of 24 showed large differences between the initial result and the repeated result which exceeded the CAP limits for allowable error. The number and rates (%) of large differences for within and outside the analytical measurement range (AMR) were 12 (2.1%) and 12 (41.4%), respectively. For the 572 critical values within the AMR for each test category, the mean absolute difference (mmol/L) and difference(%) between the two testing runs were: potassium, 0.1 mmol/L (2.7%); sodium, 2.1 mmol/L (1.7%); calcium, 0.05 mmol/L (3.0%); glucose, 0.18 mmol/L (2.6%).

**Conclusions:**

When the initial chemistry critical values are within the AMR, repeated testing does not improve accuracy and is therefore unnecessary. When the initial chemistry critical values are outside the AMR, however, the benefit of repeated testing justifies its performance and makes it necessary. Performing repeat clinical testing on a case-by-case, rather than routine, basis can improve patient care by delivering critical values more rapidly while providing savings on reagent costs associated with unnecessary repeat testing.

## Introduction

Since Lundberg [1] first described a critical value as a laboratory result that reflects a potentially life-threatening emergency in 1972, well-defined requirements for the identification, handling, documentation and auditing of laboratory critical values have been published by the Joint Commission International, ISO 15189, and the College of American Pathologists (CAP) [2]–[4]. In addition, the Chinese Hospital Association (CHA) has recently established the requirement of reporting critical values [5]. This widespread adoption of measuring critical values into clinical practice worldwide has been accompanied by a routine practice of performing repeat testing, despite a lack in empirical evidence to indicate the usefulness and necessity of repeat critical values. Correspondingly, no regulatory requirements for verification of critical value results by repeat analysis have been established.

Currently, in many laboratories, repeat testing is performed as a routine practice prior to communicating the critical laboratory values to the caregiver. The reasoning for this repeat testing routine is to ensure the accuracy of results and to avoid reporting false or erroneous data. While the repeat testing causes some delay in reporting the critical test results, and consequently delays physician intervention, it also increases the monetary and man-labor costs of the analytical processing. Indeed, the practicality of routinely verifying each critical value result by repeat analysis has been questioned in recent years [6]–[8]. A Q-Probes survey of clinical laboratories conducted by CAP found that 61% performed routine repeat testing for critical chemistry values and identified the associated delay to reporting as 10 – 14 minutes; more importantly, neither the delay to reporting nor the related wasted resources were found to provide any significant benefit in preventing analytic errors [9], [10].

To provide further empirical evidence of whether repeat testing of critical laboratory values is useful and necessary, we analyzed the accuracy of 601 repeated chemistry critical values obtained from a total of 72,259 routine clinical chemistry specimens over a 4-month period in our clinical laboratory, as compared to the allowable error limit put forth in the CAP.

## Materials and Methods

### Setting

The Clinical Chemistry Laboratory of the General Hospital of Jinan Military Region (Jinan, China) serves its approximately 2,000 bed in-patient population as well as the 0.9 million annual out-patient group, providing an annual test volume of ∼4.3 million samples. For this study, clinical samples that were submitted between January 1 and April 30 of 2013 for testing of common critical analytes (potassium, sodium, calcium, and glucose) were selected for analysis. The clinical testing of all four analytes was performed with a separating gel vacuum tube (BD Diagnostics, USA) and the UniCel DXC-800 automated analyzer (Beckman Coulter Inc., USA). After referring to the laboratory testing documentation, the repeat tests were performed either on the same analyzer unit as used for the initial run or on another DXC-800 analyzer unit using the exact parameters as in the initial run. Both the analyzers were calibrated periodically and maintained daily for quality control according to the manufacturers’ instructions. In addition, the performance of the two analyzers was compared weekly to ensure common test results.

### Ethics statement

This study was designed in accordance with the Helsinki Declaration and carried out with approval from the Ethics Committee of the General Hospital of Jinan Military Region. The local Institutional Review Board waived the need for written informed consent from participants due to the retrospective nature of the study and the ability to sufficiently anonymized the samples.

### Critical value range

Following the CHA guidelines [5] and expert recommendations, the following cut-off values were selected for the four critical analytes: potassium, <2.5 mmol/L or >6.5 mmol/L; sodium, <120 mmol/L or >160 mmol/L; calcium, <1.5 mmol/L or >3.25 mmol/L; glucose, <2.8 mmol/L or >30 mmol/L.

### Initial testing results and repeat testing study design

A total of 601 chemistry critical values obtained from 72,259 routine clinical chemistry specimens were repeat tested. These values included 255 potassium critical results (133 with <2.5 mmol/L, 122 with >6.5 mmol/L, 254 within the analytic measurement range (AMR), 1 below the AMR, and 0 above the AMR), 132 sodium critical results (103 with <120 mmol/L, 29 with >160 mmol/L, 130 within the AMR, 2 below the AMR, and 0 above the AMR), 108 calcium critical results (88 with <1.5 mmol/L, 20 with >3.25 mmol/L, 107 within the AMR, 1 below the AMR, and 0 above the AMR), and 106 glucose critical results (65 with <2.8 mmol/L, 41 with >30 mmol/L, 81 within the AMR, 0 below the AMR, and 25 above the AMR). For initial values above the AMR, the tests were repeated with diluted sample. For initial values below or within the AMR, the tests were repeated with undiluted sample.

### Data processing and analysis

All the results from initial and repeated testing were recorded in our laboratory information system (LIS). For analysis, the data were transferred to Microsoft Excel 2007. The absolute value and the percentage of difference between the two testing runs were calculated for each critical value and then compared with the allowable error limit published by the CAP. CAP recognizes at least two error limits, one used in the linearity, calibration verification surveys and the other one used during the evaluation of proficiency (PT) surveys, and we applied the CAP PT error limits. If the absolute or percentage difference between the two test runs was greater than the CAP allowable error limit, then the initial result was classified as having a “large difference”. The CAP allowable error limit, critical values, and the AMR for the four test categories are shown in Table 1.

**Table 1:** Critical Values, Allowable Error Limits, and AMRs.

Tested Analyte	Critical Values	Allowable Error Limit*	AMR#
Potassium, mmol/L	<2.5 or >6.5	±0.5	1–15
Sodium, mmol/L	<120 or >160	±4.0	100–200
Calcium, mmol/L	<1.5 or >3.25	±0.25	0.5–5.0
Glucose, mmol/L	<2.8 or >30.0	±0.3 or 10%	0.2–33.3

* Extrapolated from CAP participant surveys.

# According to the reagent manufacturers’ instructions (Beckman Coulter Inc.).

## Results

### Number and rates of identified large differences for the potassium, calcium, sodium, and glucose critical values

Of the total 601 repeated chemistry critical values, 572 were characterized as within the AMR, 25 as above the AMR, and 4 as below the AMR. Only 4.0% (24/601) showed a large difference (Table 2), so that the majority (96% of the results) showed good agreement between the initial and repeated results. The numbers and rates of large differences found among each of the four test categories samples initially classified as: within the AMR were 12 (1 calcium, 1 glucose, 3 potassium, and 7 sodium) and 2.1%; above the AMR were 11 (only 11 glucose) and 44.0%; and below the AMR were 1 (sodium) and 25.0%. Therefore, sodium had the highest (5.4%) and calcium had the lowest (0.9%) rates of large differences among the values with initial classification of within the AMR.

**Table 2:** Numbers and Rates of Repeat Tests Showing Large Difference*.

Tested Analyte	Initial, n	Within the AMR		Above the AMR		Below the AMR	
		Repeats, n	Large Difference, n(%)	Repeats, n	Large Difference, n(%)	Repeats, n	Large Difference, n(%)
Potassium	255	254	3(1.2)	0	0(0)	1	0(0)
Sodium	132	130	7(5.4)	0	0(0)	2	1(50.0)
Calcium	108	107	1(0.9)	0	0(0)	1	0(0)
Glucose	106	81	1(1.2)	25	11(44.0)	0	0(0)
Total	601	572	12(2.1)	25	11(44.0)	4	1(25.0)

* The percentage of repeats for which the difference between the initial and repeated values exceeded the CAP limits for allowable error.

### Absolute and percentage of difference between the two runs for samples initially classified as within the AMR

Table 3 shows the mean absolute difference between the two testing runs for the 572 critical values initially classified as within the AMR for each test category and for subgroups of low and high critical value. The lowest mean percentage of difference (1.4%) was represented by the sodium high critical value subgroup, while the highest mean percentage of difference (3.5%) was represented by the potassium low critical value subgroup.

**Table 3:** Mean Absolute and Percentage of Difference between the Two Runs for Specimens Originally Classified as Within the AMR.

Tested Analyte	Subgroups	Specimens, n	Result Range	Result Mean	Absolute Difference	
					Mean	Percentage
Potassium, mmol/L	All specimens	254	1.26–9.71	4.60	0.10	2.72
	<2.5	132	1.26–2.61	2.18	0.08	3.47
	>6.5	122	6.28–9.71	7.20	0.14	1.90
Sodium, mmol/L	All specimens	130	100.2–181.5	126.6	2.1	1.70
	<120	101	100.2–124.1	114.9	2.1	1.80
	>160	29	158.1–181.5	167.2	2.3	1.35
Calcium, mmol/L	All specimens	107	0.61–4.13	1.70	0.05	3.02
	<1.5	87	0.61–1.58	1.29	0.04	3.25
	>3.25	20	3.15–4.13	3.50	0.07	2.00
Glucose, mmol/L	All specimens	81	0.35–33.15	7.90	0.18	2.56
	<2.8	65	0.35–2.81	2.02	0.05	2.62
	>30.0	16	29.16–33.15	31.80	0.74	2.31

### Features of the large differences

Table 4 shows the features of the 24 large difference critical values, including the absolute differences for each of the four categories.

**Table 4:** Features of the Large Difference.

Tested Analyte	Total specimens, n	Large Difference, n(%)	Allowable Error Limit*	Run 1	Run 2	Mean	Absolute Difference	Percentage of Difference
Potassium, mmol/L	255	3(1.2)	±0.5	2.49	3.00	2.74	0.51	18.58
				6.98	6.44	6.71	0.54	8.05
				9.10	8.57	8.84	0.53	6.00
Sodium, mmol/L	132	8(6.1)	±4.0	98.7	102.9	100.80	4.20	4.17
				105.9	100.2	103.05	5.70	5.53
				114.4	120.5	117.45	6.10	5.19
				118.1	122.3	120.20	4.20	3.49
				119.9	124.1	122.00	4.20	3.44
				164.5	160.4	162.45	4.10	2.52
				176.0	180.9	178.45	4.90	2.75
				181.5	174.6	178.05	6.90	3.88
Calcium, mmol/L	108	1(0.9)	±0.25	3.92	3.65	3.78	0.27	7.13
Glucose, mmol/L	106	12(11.3)	±0.3 or 10%	1.83	1.52	1.68	0.31	18.51
				44.86	52.98	48.92	8.12	16.60
				44.88	51.70	48.29	6.82	14.12
				47.97	55.67	51.82	7.70	14.86
				50.11	60.97	55.54	10.86	19.55
				52.85	64.62	58.735	11.77	20.04
				61.53	77.22	69.375	15.69	22.62
				63.46	80.87	72.165	17.41	24.13
				66.32	83.11	74.715	16.79	22.47
				67.63	84.24	75.935	16.61	21.87
				68.30	87.10	77.70	18.80	24.20
				70.72	96.30	83.51	25.58	30.63

* Extrapolated from CAP participant surveys.

## Discussion

Repeat testing of analytes with critical values is generally performed to confirm the result’s accuracy so as to avoid basing critical care decisions on false or erroneous results [6]–[7]. In the early days of laboratory automation, this procedure offset the insufficiencies of the testing instrumentation, which lacked highly-sensitive sensors as well as (fibrin) clot detectors. In addition, LIS technology was in its infancy at that time and issues of sample misiden­tification and insufficient specimen volume were common. The substantial technological advances in clinical testing that have been developed and introduced into routine clinical practice over the past decade have overcome these challenges; for example, specimen barcoding and two-way communication between the LIS and the instrumentation have helped to ensure proper patient-sample identification and ultra­sensitive sensors and clot detectors have improved precision remarkably. These advances have likely made the routine processing of repeat measurements unneces­sary, although the process may continue to be useful in certain circumstances, such as to address specimens yielding questionable or infeasible results on first run and delta failures [8].

In a study by Chima et al. [6], a total of 580 repeated tests for potassium, glucose, platelet count (PLT), or activated partial thromboplastin time (APTT) were evaluated for differences in accuracy between the initial and repeat test results; the findings indicated that the repeat testing is largely unnecessary, with >95% of the repeated values being within the acceptable limits of difference. Ultimately, the authors concluded that the differences in repeat values did not change the eventual treatment protocol selected by the clinicians. Similarly, in a review of 2,627 specimens (498 hemoglobin (HGB) critical results, 493 white blood cell (WBC) critical results, 551 PLT critical results, 533, prothrombin time (PT) critical results, and 552 APTT critical results), Toll et al. [7] found that the repeat testing yielded results for 99% of the specimens were within their laboratory’s acceptable tolerance Limits for repeat runs (ATLRs); the authors also concluded that, in general, repeated testing for critical values did not offer an advantage or provide additional benefit in hematology and coagulation settings. Neither of these two studies examined the effects on time delay to reporting the critical values to the treating physician, nor did they evaluate the between-tests differences in the critical values from specimens with regard to the AMR character (ranging from within the AMR to outside the AMR).

In another study by Deetz et al. [8], 25,553 repeat laboratory values from 30 common chemistry tests (six types of electrolytes, three types of drugs, four types of immunoassay-detected analytes, three types of arterial blood gases, and 14 other routine clinical chemistry analytes) yielding a total of 855,009 results were evaluated for differences from the CAP allowable error limit. Large differences (initial value vs. repeat value) were found for 2.6% of all repeated tests. Moreover, of these 668 errors, only 102 (electrolytes, n = 1; drugs, n = 2; immunoassay-detected analytes, n = 0; arterial blood gases, n = 52; routine clinical chemistry analytes, n = 47) represented specimens with initial values that fell within the AMR (0.5% of all repeated values). These findings suggest that when initial results are within the AMR, as obtained by automated chemistry testing techniques, the repeated testing is unnecessary and may only serve to delay the reporting of result and critical care decisions. In addition, this study had also examined the median delays in reporting associated with their evaluated critical values and found that the delays ranged from 5 (blood gases) to 17 (glucose) minutes – substantial amounts of time in urgent care settings. The results from another study conducted by CAP, which had surveyed 40 critical test results from four test types performed at 86 clinical laboratories, indicated that 61% of the laboratories always repeat testing of critical results and that the procedure-related median delay could be up to 17 – 21 minutes [9], [10].

The Clinical Chemistry Laboratory of the General Hospital of Jinan Military Region has performed routine repeat testing of critical values for more than 10 years. The standard protocol includes comparison of the initial and repeated results; when they are in agreement, the initial value is deemed to have been verified, but if the difference between the initial and repeated results exceeds the CAP limits for allowable error, then the test is performed a third time and the average of the two results that are in closest agreement is reported to the treating physician. However, based on the accumulated findings from the previous studies cited above, we began to question the utility and necessity of routine repeat testing for clinical chemistry critical values and whether the practice was actually negatively impacting the timelines of our critical care. Ultimately, the new result from our focused investigation, presented herein, agreed with those from the previous studies. Specifically, repeat testing was shown to be unnecessary in the majority of cases, likely due to the highly sensitive and precise instrumentation used for the initial testing, and indicated that the benefit of repeat testing may lie principally in cases when the initial results falls beyond the AMR.

Our finding of large differences for repeat testing of specimens with initial results below the AMR may be due to “short sampling” or other preanalytic or analytic error [8]. In general, however, we noted that specimens providing initial results above the AMR required dilution to obtain a more accurate final result. Finally, we observed that the absolute or percentage differences between the duplicate runs were most frequently greater than the CAP allowable error. Taken together, these findings indicate that when initial results are outside the AMR then repeat testing is necessary, but when the initial results of clinical chemistry critical values are within the AMR then the mean absolute difference and percentage of difference between the first and second analyses are not clinically significant and there is no need to check values again for accuracy. It is important to note, here, that our study focused solely on four critical chemistry analytes (potassium, sodium, calcium and glucose); like the previously published findings of Chima et al. [6] and Toll et al. [7] our current findings are only directly applicable to a certain group of analytes and testing protocol. The utility and necessity of repeat testing for other analytes (critical or not) or using other methods (such as immunoassay) will need to be addressed by other studies.

The definition of what constitutes a significant difference between the initial and repeated values has been variable throughout the literature, with definitions ranging from biologic variability [11], subjective opinion, clinician survey consensus, or regulatory requirements; ultimately, this study-to-study variability may have led to different results designated as “large differences” in the reported studies. In the current study, we applied the CAP allowable error limits because they are recognized and applied over a broad range of countries and medical institutes. Certainly, as suggested by Deetz et al. [8], some of the CAP criteria are questionable; for example, the criterion for sodium is ±4 mmol/L (∼±2.8%), whereas the criterion for calcium is ±0.25 mmol/L (∼±10.0%). It could be argued that the former is clinically insignificant, whereas the latter is clinically significant. Accordingly, this may have impacted our study’s findings and may explain why there was only one large difference found for the calcium analyte but eight for the sodium analyte. Similarly, the reasons for the higher amount of specimens with large difference of the sodium analyte remain unclear and future prospective evaluation may help to further understand the implications of this distinctive finding.

## Conclusion

Our observations are in agreement with those of Deetz et al. [8], indicating that when the initial chemistry critical values for potassium, calcium, sodium, and glucose are within the AMR then repeat testing does not offer better accuracy or provide additional benefit, making it an unnecessary process. However, when these initial chemistry critical values fall outside the AMR then repeat testing is necessary. By performing repeat testing of critical values on a case-by-case, rather than routine, basis can improve patient care by delivering critical values more rapidly and can potentially save monetary and man-power costs. These findings, however, must be taken with caution, as our study was relatively small and relied on data from a single laboratory and it is known that the difference rates of critical values may vary depending on instrumentation and other variables of individual laboratories, such as internal quality assurance practices. Therefore, the necessity of repeat critical values needs to be further confirmed by larger studies using more heterogeneous datasets in the future.
